# Lymphocyte Activation Gene-3 (LAG-3) Negatively Regulates Environmentally-Induced Autoimmunity

**DOI:** 10.1371/journal.pone.0104484

**Published:** 2014-08-14

**Authors:** Vibha Jha, Creg J. Workman, Tracy L. McGaha, Liping Li, Jaya Vas, Dario A. A. Vignali, Marc Monestier

**Affiliations:** 1 Temple Autoimmunity Center and Department of Microbiology and Immunology, Temple University School of Medicine, Philadelphia, Pennsylvania, United States of America; 2 Department of Medicine, Section of Rheumatology, Temple University School of Medicine, Philadelphia, Pennsylvania, United States of America; 3 Department of Immunology, St. Jude Children’s Research Hospital, Memphis, Tennessee, United States of America; Uniform Services University of the Health Sciences, United States of America

## Abstract

Environmental factors including drugs, mineral oils and heavy metals such as lead, gold and mercury are triggers of autoimmune diseases in animal models or even in occupationally exposed humans. After exposure to subtoxic levels of mercury (Hg), genetically susceptible strains of mice develop an autoimmune disease characterized by the production of highly specific anti-nucleolar autoantibodies, hyperglobulinemia and nephritis. However, mice can be tolerized to the disease by a single low dose administration of Hg. Lymphocyte Activation Gene-3 (LAG-3) is a CD4-related, MHC-class II binding molecule expressed on activated T cells and NK cells which maintains lymphocyte homeostatic balance via various inhibitory mechanisms. In our model, administration of anti-LAG-3 monoclonal antibody broke tolerance to Hg resulting in autoantibody production and an increase in serum IgE level. In addition, LAG-3-deficient B6.SJL mice not only had increased susceptibility to Hg-induced autoimmunity but were also unresponsive to tolerance induction. Conversely, adoptive transfer of wild-type CD4^+^ T cells was able to partially rescue LAG-3-deficient mice from the autoimmune disease. Further, in LAG-3-deficient mice, mercury elicited higher amounts of IL-6, IL-4 and IFN-γ, cytokines known to play a critical role in mercury-induced autoimmunity. Therefore, we conclude that LAG-3 exerts an important regulatory effect on autoimmunity elicited by a common environmental pollutant.

## Introduction

Mercury (Hg) is a hazardous environmental contaminant. Several studies report that mercury exposure is associated with autoimmune dysfunction in occupationally-exposed humans [1]–[5]. In susceptible H2^S^ mice such as A.SW or B6.SJL, subtoxic levels of HgCl_2_ induce an autoimmune dysfunction characterized by glomerulonephritis, production of antinucleolar autoantibodies (ANoA) and hypergammaglobulinemia (especially pronounced for IgE and IgG1) [6]–[11]. The increase in polyclonal immunoglobulins peaks 2 weeks after the first HgCl_2_ injection and returns to normal levels by week 4. The production of antigen-specific ANoA starts at week 2 and continues to increase for about 4 to 6 weeks. Susceptible animals can however be tolerized to Hg. When A.SW mice receive a single low dose injection of HgCl_2_, they become resistant to a subsequent standard challenge of HgCl_2_ [12]. Administration of Hg can also potentiate disease in other mouse models of autoimmunity [13], [14]. The mechanisms by which mercury can induce disease are not fully understood [7] although interference with signal transduction pathways in T cells might play an important role [15].

Lymphocyte activation gene-3 (LAG-3) is a type I transmembrane protein expressed on activated CD4^+^ and CD8^+^ T cells, a subset of γδ T cells, NK cells and regulatory cells (Tregs) [16], [17]. LAG-3 has a genomic proximity to CD4 [18] and like CD4, it binds to MHC-II [17], [19] albeit with a higher affinity [20]. The functions of LAG-3 are dependent on its interaction with MHC-II and a conserved KIEELE motif present in its cytoplasmic domain [16].

LAG-3-deficient mice do not exhibit any adverse phenotype [16]. In fact, the initial analysis of LAG3−/− did not reveal a defect in T cell function and no gross T cell abnormalities are present [21], [22]. However, LAG-3-deficient T cells undergo an increase in expansion as compared to the wild-type T cells in a lymphopenic environment. Additionally, *Lag-3^−/−^* mice have increased numbers of lymphocytes that do not normally express LAG-3 such as B cells, granulocytes, macrophages and DCs indicating that deregulation of LAG-deficient T cells influences the expansion of cell types from other lineages [23].

A real-time PCR study indicates that antigen-specific CD4^+^ T cells of regulatory phenotype have higher expression of LAG-3 as compared to the antigen-specific effector CD4^+^ T cells. Both natural and induced Tregs lacking LAG-3 *in vivo* are defective in their suppressive activities [23]. Also, ectopic expression of LAG-3 imparts regulatory phenotype to CD4^+^CD25^−^ T cells [23], [24]. In addition, Liang et al. have reported that LAG-3 on Tregs can engage MHC class II on DCs and induce an ITAM-mediated inhibitory signaling pathway to suppress DC maturation and function [25].

Regulation of homeostatic balance and maintenance of tolerance are two critical immunological processes that prevent the development of autoimmune diseases. To understand the impact of homeostatic balance on environmentally-induced autoimmunity, we sought to investigate the role of LAG-3 in mercury-induced autoimmunity. Our observations indicate that mice exposed to Hg have higher expression of LAG-3 on CD4^+^ T cells. Abrogation of LAG-3 functions, either by administering anti-LAG-3 monoclonal antibody or by genetic ablation of LAG-3, results in an increased susceptibility to mercury-induced autoimmune disease. Thus our data indicate that LAG-3 plays an important role in the prevention of environmentally-induced autoimmunity.

## Materials and Methods

### Mice

A.SW/Snj (H2^S^) were obtained from Jackson Laboratories (Bar Harbor, ME). *Lag-3^−/−^* C57BL/6 (H2^b^) mice were described previously and were fully backcrossed onto the B6 background [22]. *Lag-3^−/−^* C57BL/6 mice were crossed with B6.SJL (H2^S^) mice to generate *Lag-3^−/−^* B6.SJL mice. Homozygosity for LAG-3 targeted deletion and for H2^S^ was determined by PCR as described before [26], [27]. All mice used for the experiments were at least 2 months old. Animals were sacrificed using a CO_2_ chamber.

### Ethics Statement

We followed the regulations under all federal, state and local laws while using the animals. The experiments using animals were performed with approval and oversight of Temple University, School of Medicine Institutional Animal Care and Use Committee under protocol number 925.

### Data Availability Statement

The authors confirm that data corresponding to the findings are completely available without restriction. All data are included in the manuscript.

### Serum Collection

Mice were anesthetized using regulated levels of isoflurane and then bled once a week retro-orbitally for up to 4 weeks after the start of treatments. The blood samples were incubated at 37°C for 45 minutes, followed by incubation at 4°C for 2 hours. Coagulated blood was removed using a wood applicator. Samples were then centrifuged at 8000 rpm for 5 minutes. The separated serum was transferred to 0.65 ml eppendorf tubes and stored at −20°C for future analysis.

### HgCl_2_ and antibody treatment

Mercury-induced autoimmunity was induced in groups of 5 or 6 mice according to a standard protocol by sub-cutaneous (s.c.) injections given on days 0, 2 and 4 (each injection consisted of 30 µg of HgCl_2_ in 100 µl of sterile PBS). Tolerance was induced by a single intraperitoneal (i.p.) injection of 3 µg of HgCl_2_ in 300 µl of sterile PBS given on day -7 [12]. In addition to HgCl_2_, some groups of mice also received i.p. injections of blocking anti-LAG-3 monoclonal antibody [28]. An irrelevant rat IgG was used as control. Antibodies were administered in 300 µl of sterile PBS.

### ANOA Immunofluorescence

Serum IgG1 and IgG2a ANoA (the main isotypes produced during mercury-induced autoimmunity) levels were determined by indirect immunofluorescence (IF) as described previously [29]. Sera diluted in PBN (PBS containing 1% BSA and 0.02% sodium azide) were incubated with HEp-2 slides (Antibodies, Inc, Davis, CA) for 30 minutes and ANoA were detected with FITC-conjugated goat anti-mouse IgG1 or IgG2a antibodies (Southern Biotechnology Associates, Birmingham, AL). The inverse of the highest serum dilution at which nucleolar fluorescence could be detected was defined as the ANoA titer.

### ELISA for serum Ig

Total serum IgG1 and IgE levels were detected using a sandwich ELISA as described previously [29]. Briefly, 96 well polyvinylchloride plates (BD Biosciences, San Jose, CA) were coated with goat anti-mouse Ig ĸ (Southern Biotechnology Associates, Birmingham, AL) or rat anti-mouse IgE (Clone R35-72, BD Pharmingen, San Diego, CA). Plates were then blocked for 30 minutes at room temperature (RT). Sera samples diluted 1 in 50,000 for IgG1 or 1 in 100 for IgE were added and incubated at RT. IgG1 was detected using alkaline phosphatase (AP)-coupled goat anti-mouse IgG1 (Southern Biotechnology Associates, Birmingham, AL). IgE was detected using biotinylated rat anti-mouse IgE (Clone, R35-72, BD Pharmingen, San Diego, CA). Antibody levels in the samples were extrapolated from a standard curve generated using varying concentrations (3.15–400 µg/ml) of ASWU1 (IgG1) previously purified in our laboratory [30] or purified mouse IgE (IgE-3) (BD Biosciences, San Jose, CA).

### Cytokine ELISA

For cytokine ELISA, splenocytes were cultured in complete RPMI 1640 medium in 24 well plates coated with 3 µg/ml of anti-mouse CD3 and 2 µg/ml of anti-mouse CD28. Supernatants from cultured cells were collected at different time points to perform cytokine ELISA. Total IL-4, IL-6 and IFN-γ were measured by sandwich ELISA using reagents (recombinant mouse cytokine, anti-cytokine antibodies and biotinylated anti-cytokine antibodies) purchased from BD Pharmingen (San Diego, CA).

### ELISA for soluble LAG-3

This ELISA was performed as described earlier [31].

### Detection of immune complex deposits in kidney

Four weeks after the first HgCl_2_ injection, kidneys were harvested from mice and embedded in Tissue-Tek OCT compound (Sakura, Torrance, CA) and snap-frozen on dry ice. A cryostat microtome was used to cut 4 µm sections. The sections were air dried, fixed with cold acetone and stained with goat anti-mouse IgG FITC (Southern Biotechnology Associates, Birmingham, AL) to detect IgG deposits. Glomerulonephritis was scored in a blinded fashion using 1–4 scale based on the percentage of glomeruli affected. The grading scale was 0 =  no glomerulonephritis, 1+ ≤15%, 2+16–40%, 3+41–70%, and 4+ >70% of glomeruli affected. The lesions graded included thickening of the mesangium, noticeable increases in both mesangial and glomerular cellularity with/without superimposed inflammatory exudates and capsular adhesions, glomerulosclerosis, and cast formation. Scoring was performed on at least 200 glomeruli in a 40× field per kidney [32]. Immune complex deposition in kidneys was quantified by blinded observers and arbitrarily scored from 0 to 4 dependent on the intensity and amount of the anti-IgG staining in the glomeruli [32].

### Flow Cytometry

To detect CD4^+^ T cells expressing LAG-3, splenocytes were subjected to RBC lysis and then stained with PE-conjugated anti-LAG-3 monoclonal antibody and FITC-labeled rat anti-mouse CD4 (BD Biosciences, San Jose, CA). LAG-3^+^ T cells were then identified by using a BD FACSCalibur (BD Biosciences, San Jose, CA). For the adoptive transfer experiments, splenocytes were harvested from naïve wild-type and *Lag-3^−/−^* mice and RBC lysis was carried out using 0.164 M NH4Cl. Cells were then stained with FITC–labeled rat anti-mouse CD4 (BD Biosciences, San Jose, CA). CD4^+^ T cells were sorted using a DakoCytomation MoFlo (DakoCytomation, Inc., Fort Collins, CO). Flow cytometry data were analyzed using FlowJo software (Tree Star, Ashland, OR).

### Statistical methods

Graph Pad prism software was used to generate figures and perform statistical analyses. The method of stastical analysis used for each experiment is described in the corresponding figure legend.

## Results

### Exposure to Hg results in higher expression of LAG-3 on T cells and in an increased amount of soluble LAG-3 in serum

**Figure 1:**
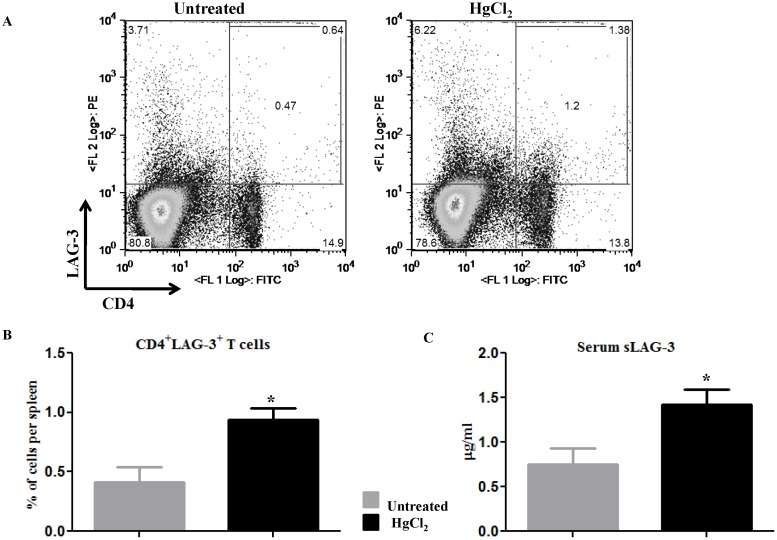
Exposure to Hg results in higher expression of LAG-3 on CD4^+^T cells and in an increased amount of soluble LAG-3 in serum. (A) B6.SJL mice were left untreated or given 3 injections of HgCl_2_ s.c. on days 0, 2 and 4. Splenocytes were harvested on day 8 and analyzed by flow cytometry. Figure depicts a representative dot plot of total splenocytes gated for LAG-3^+^ CD4^+^ T cells. (B) Results are expressed as frequency of LAG-3^+^ CD4^+^ T cells in each group (n = 3 or 4). (C) A.SW mice were challenged with 3 injections of HgCl_2_ (30 µg/mouse) on days 0, 2 and 4. Concentration of soluble LAG-3 (sLAG-3) in serum was measured by ELISA (n = 4 or 5). Unpaired t test was used for statistical analysis where *indicates *p*<0.05.

Exposure of susceptible strains of mice to subtoxic levels of mercury induces proliferation and activation of CD4^+^ T cells [33], [34]. Because LAG-3 is expressed on activated T cells, we decided to investigate the effect of HgCl_2_ treatment on the expression of LAG-3. B6.SJL mice were either given three injections of HgCl_2_ on days 0, 2 and 4 or were left untreated. On day 8 splenocytes were stained with anti-CD4 and anti-LAG-3 monoclonal antibodies. Expression of LAG-3 on CD4^+^ T cells is shown in Fig. 1A and Fig. 1B. Exposure of mice to HgCl_2_ resulted in a two-fold increase in the percentage of CD4^+^ cells expressing LAG-3.

LAG-3 undergoes proteolytic cleavage by two metalloproteases to release a 54 KDa molecule called soluble LAG-3 (sLAG-3) which contains all of its extracellular domains [31], [35]. Both *in vitro* and *in vivo* T cell activation results in an increase in the production of sLAG-3 [31]. Release of sLAG-3 is essential for proliferation of T cells and inactivation of this process results in inhibition of T cell multiplication and cytokine production [34]. This prompted us to check the level of serum sLAG-3 in mice challenged with HgCl_2_. A.SW mice received 3 injections of HgCl_2_ on days 0, 2 and 4. As depicted in Fig. 1C, the serum sLAG-3 concentration was 1.42 µg/ml after one week of Hg treatment versus 0.75 µg/ml in the untreated group. These experiments showed that exposure of mice to HgCl_2_ causes an increase in LAG-3^+^ CD4^+^ T cells and in the concentration of sLAG-3 in serum thus suggesting a possible role for LAG-3 in the T cell-mediated immune response to mercury.

### LAG-3-deficient B6.SJL mice display an increased susceptibility to mercury-induced autoimmunity

**Figure 2:**
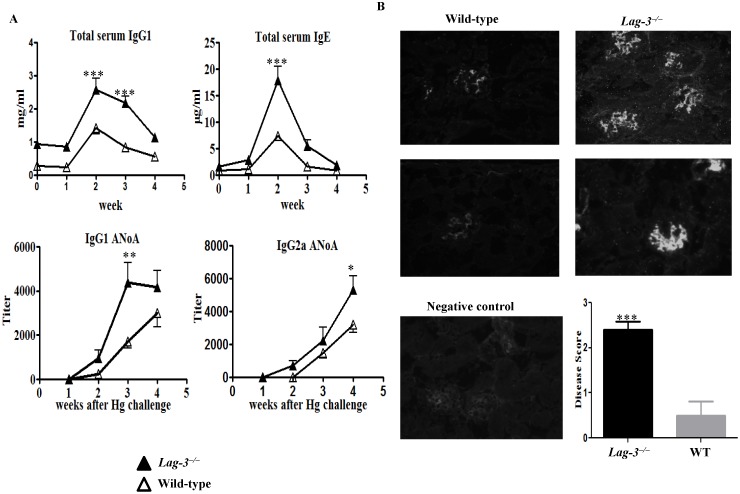
LAG-3-deficient B6.SJL mice display an increased susceptibility to mercury-induced autoimmunity. (A) Wild-type or *Lag-3^−/−^* B6.SJL mice were challenged with 3 weekly s.c. injections of 30 µg HgCl_2_/mouse during four weeks for a total of 12 injections. Serum Ig and autoantibodies were measured as described in the Material and Method section (n = 5 or 6). The levels of Ig depicted at week 0 represent the baseline levels in untreated LAG-3−/− and WT mice whereas the untreated mice in both groups had no detectable autoantibodies. (B) Mice from this experiment were sacrificed after 4 weeks. Their kidney sections were stained with goat anti-mouse IgG-FITC to detect IgG deposits (n = 5). Negative control depicts the staining of kidney section obtained from 3 week old untreated B6.SJL mice. The extent of kidney disease was scored depending on the amount and intensity of IgG staining as described in details in Material and Method section. To determine statistical significance between the groups of fig. 2A and 2B two way ANOVA was used where *indicates *p*<0.05, **indicates *p*<0.005 and ***indicates *p*<0.0001.

B6.SJL is mouse strain congenic to C57BL/6 which possesses the Hg-susceptible H2^S^ haplotype instead of the original H2^d^. This strain allows for cross-breeding of various targeted mutations which are typically present on the same B6 background. We thus evaluated mercury-induced autoimmunity in LAG-3-deficient B6.SJL mice. The LAG-3 mutation was bred onto B6.SJL mice expressing the susceptible H2^S^ MHC haplotype. Wild-type or *Lag-3^−/−^* B6.SJL mice were given 3 s.c. injections of HgCl_2_ per week for 4 weeks up to a total of 12 injections. As shown in Fig. 2A, the *Lag-3^−/−^* B6.SJL mice had significantly higher levels of both serum IgG1 and IgE antibodies on day 14 and they developed higher titers of IgG1 and IgG2a ANoA. The wild-type B6.SJL mice displayed a milder disease with low levels of serum polyclonal IgG1, IgE and lower titers of ANoA as compared to *Lag-3^−/−^* mice.

Since deposition of immune complexes in kidneys takes place during Hg-induced autoimmune disease, we performed immunofluorescence staining of kidney sections with anti-IgG FITC. The staining revealed that the *Lag-3^−/−^* mice had significantly increased immune complex deposits in their kidneys and the cumulative disease score was higher in *Lag-3^−/−^* mice as compared to the wild-type (Fig. 2B).

### A short course of HgCl_2_ injections is sufficient to induce mercury-induced autoimmune disease in LAG-3-deficient B6.SJL mice

**Figure 3:**
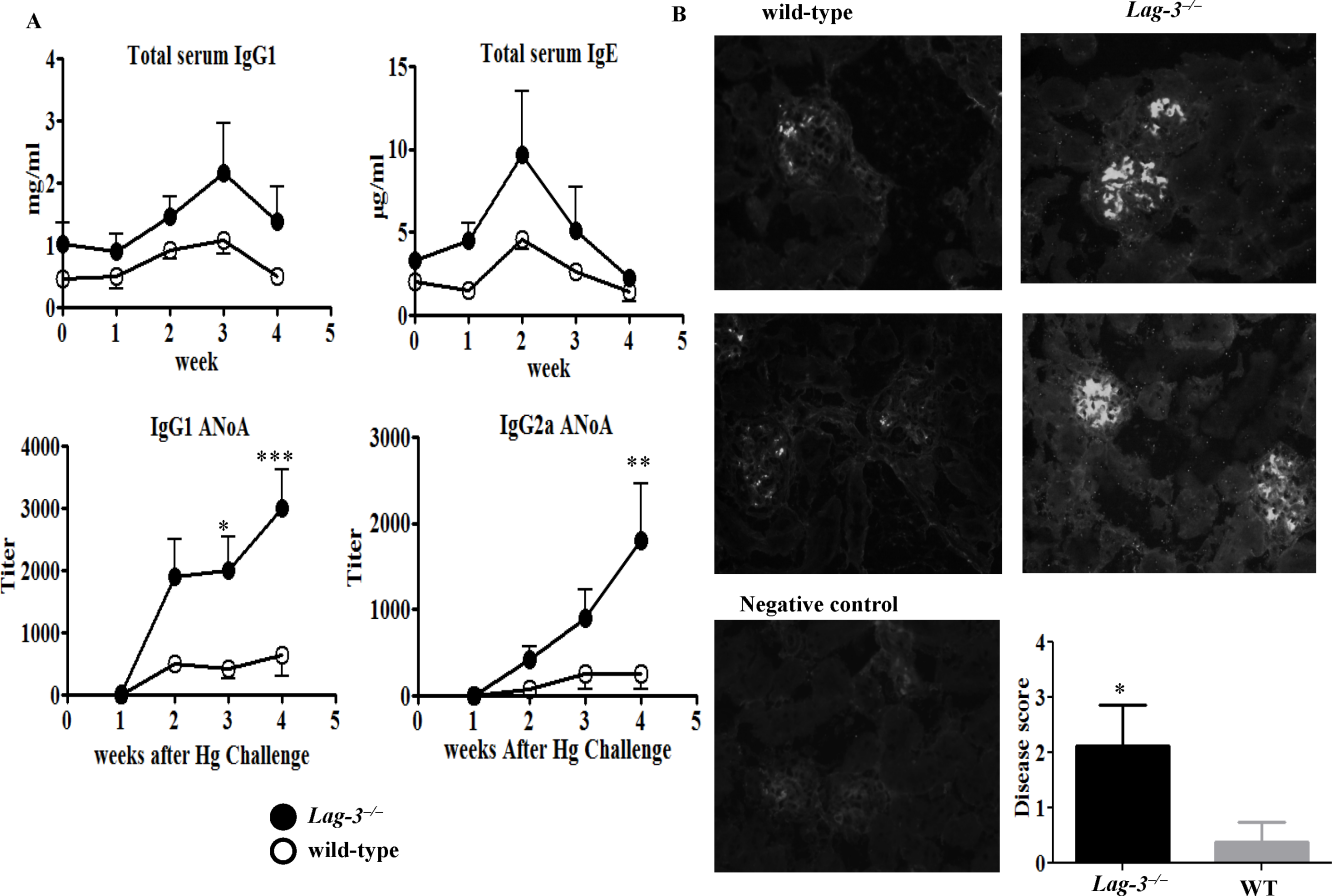
A short course of HgCl_2_ injections is sufficient to induce mercury-induced autoimmunity in LAG-3-deficient B6.SJL mice. (A) Wild-type or *Lag-3^−/−^* B6.SJL mice were challenged with HgCl_2_ (30 µg/mouse s.c.) on days 0, 2 and 4. Serum was analyzed by ELISA to determine IgG1 and IgE polyclonal antibodies and ANoA titers were measured by IF assay (n = 5 or 6). (B) Kidneys of mice from this experiment were harvested after 4 weeks, sectioned and stained with goat anti-mouse IgG-FITC to detect IgG deposit. The extent of kidney disease was scored depending on the amount and intensity of IgG staining as described in details in Material and Method section. To determine statistical significance between the groups of fig. 2A and 2B two way ANOVA was used where *indicates *p*<0.05, **indicates *p*<0.005 and ***indicates *p*<0.0001.

Unlike A.SW mice which are highly susceptible to Hg-induced autoimmunity, B6.SJL mice are moderately susceptible to Hg and develop mild manifestations if they receive only 3 injections of HgCl_2_. Hence, we hypothesized that genetic ablation of LAG-3 in B6.SJL mice would increase the susceptibility to a course of 3 injections of HgCl_2_. Therefore, *Lag-3^−/−^* or wild-type B6.SJL mice were given a challenge dose of HgCl_2_ on days 0, 2 and 4. We observed that *Lag-3^−/−^* mice developed a greater concentration of serum IgG1 and IgE immunoglobulins than the wild-type controls although the differences did not reach statistical significance. However, LAG-3-deficient mice exhibited a significantly higher titer of ANoA of both IgG1 and IgG2a isotypes, while the wild-type mice had barely detectable titers of ANoA (Fig. 3A). Likewise, *Lag-3^−/−^* mice had increased amounts of renal immune complex deposits relative to the wild-type mice (Fig. 3B).

### LAG-3-deficient mice cannot be tolerized to mercury

**Figure 4:**
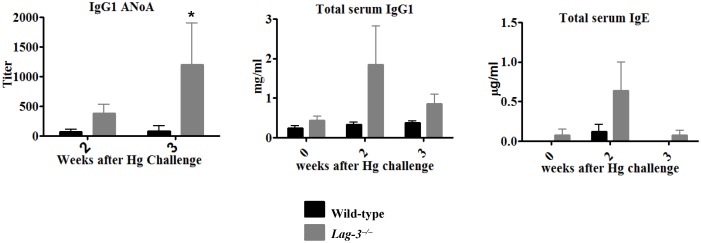
LAG-3-deficient mice cannot be tolerized to mercury. Wild-type or LAG-3-deficient mice were given a tolerogenic dose of HgCl_2_ (3 µg/mouse given i.p.) on day -7. Mice were then exposed to 3 injections of HgCl_2_ (30 µg/mouse given s.c) on days 0, 2 and 4. Serum Ig were measured by ELISA and ANoA titers were determined by IF assay (n = 5 or 6). To determine statistical significance between the groups two way ANOVA was used where *indicates *p*<0.05.

In previous studies we showed that Hg-susceptible mice can be tolerized via a single low-dose injection of HgCl_2_ [10]. Therefore, we wanted to ascertain whether LAG-3-deficient B6.SJL mice could be tolerized to Hg as well. We gave a tolerogenic dose of HgCl_2_ (3 µg/mouse) to *Lag-3^−/−^* or wild-type B6.SJL mice 1 week before they were challenged with 3 s.c. injections of HgCl_2_ on days 0, 2 and 4. As displayed in Fig. 4, we observed that tolerance was established in wild-type mice since their levels of IgG1 and IgE immunoglobulins did not increase and they produced very low titers of IgG1 ANoA after Hg challenge. In contrast the *Lag-3^−/−^* mice exhibited increased amounts of both IgG1 and IgE antibodies and they responded with high titers of IgG1 ANoA. Hence, absence of LAG-3-mediated signaling events leads to abrogation of tolerance induction by a single low dose of HgCl_2_.

### Anti-LAG-3 monoclonal antibody breaks established tolerance to mercury-induced autoimmunity

**Figure 5:**
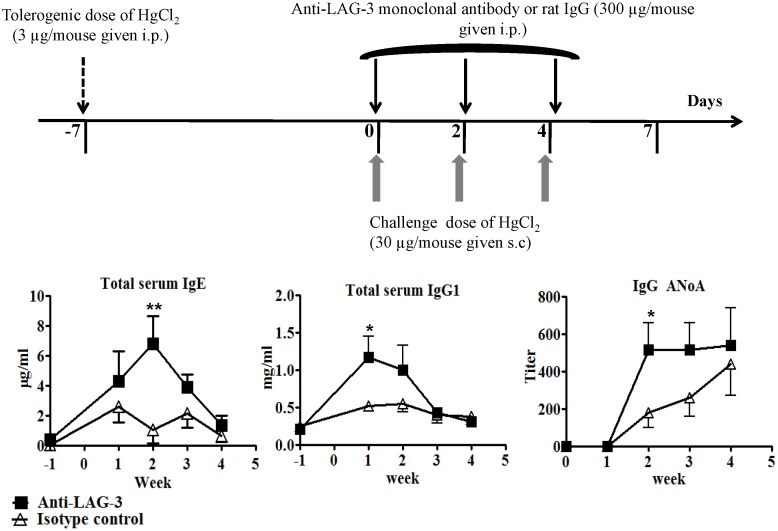
Anti-LAG-3 monoclonal antibody breaks established tolerance against mercury-induced autoimmunity. A.SW mice were tolerized to Hg and 7 days later they received a challenge dose of Hg in conjunction with anti-LAG-3 or control antibody as depicted in the Figure. Serum IgG1 and IgE levels were measured by ELISA and autoantibody titers were determined by IF assay (n = 5). The IgG and IgE concentrations depicted at wk -1 represent the baseline levels of antibodies in serum of untreated mice and no autoantibodies were detectable in untreated mice. To determine statistical significance between the groups two way ANOVA was used where *indicates *p*<0.05.

We used a LAG-3-specific monoclonal antibody (mAb) to block the functions of LAG-3 in A.SW mice to confirm whether inhibition of LAG-3 functions interferes with tolerance induction in susceptible mice and to rule out the possibility of a developmental defect due to LAG-3-deficiency. Moreover, the use of A.SW mice allowed us to monitor the effects of LAG-3 inhibition in mice with a genetic background different from B6.SJL. In mice that were previously tolerized to Hg, concomitant administration of anti-LAG-3 with HgCl_2_ challenge resulted in a loss of tolerance manifested by increased serum IgG1 and IgE levels and greater ANoA titers than in mice receiving control rat IgG (Fig. 5). Therefore, these data are in agreement with the experimental results obtained with LAG-3-deficient animals.

### LAG-3-deficient mice produced higher amounts of cytokine following mercury treatment

**Figure 6:**
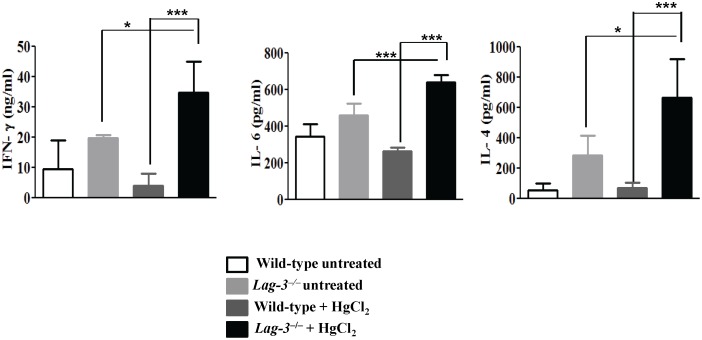
LAG-3-deficient mice produce higher amounts of cytokine following mercury treatment. Wild-type and *Lag-3^−/−^* mice were either given a challenge dose of HgCl_2_ (30 µg/mouse s.c. on days 0, 2 and 4) or were left untreated. Splenocytes were harvested on day 8 and then stimulated in vitro with plate bound anti-CD3 and anti-CD28 antibodies. Supernatant were collected after 72 hrs to determine the levels of cytokine by ELISA (n = 3 or 4). Two way ANOVA was used to determine the statistical significance between the groups where *indicates *p*<0.05 and ***indicates *p*<0.0001.

The previous experiments show that LAG-3 can modulate the regulation of Hg-induced autoimmunity. To investigate further the mechanisms by which LAG-3 influences the autoimmune disease, we compared the cytokine profile of LAG-3-deficient and wild-type B6.SJL mice which received 3 s.c. injections of HgCl_2_ on days 0, 2 and 4 or were left untreated. Splenocytes were harvested on day 8 and stimulated with anti-CD3 and anti-CD28 antibodies and cytokines in the supernatant were measured after 3 days. The *Lag-3^−/−^* mice exposed to HgCl_2_ produced significantly higher concentrations of IFN-γ, IL-4 and IL-6 as compared to both untreated *Lag-3^−/−^* mice and wild-type mice challenged with HgCl_2_ (Fig. 6).

### Transfer of wild-type CD4^+^ T cells decreases autoimmunity in LAG-3-deficient mice

**Figure 7:**
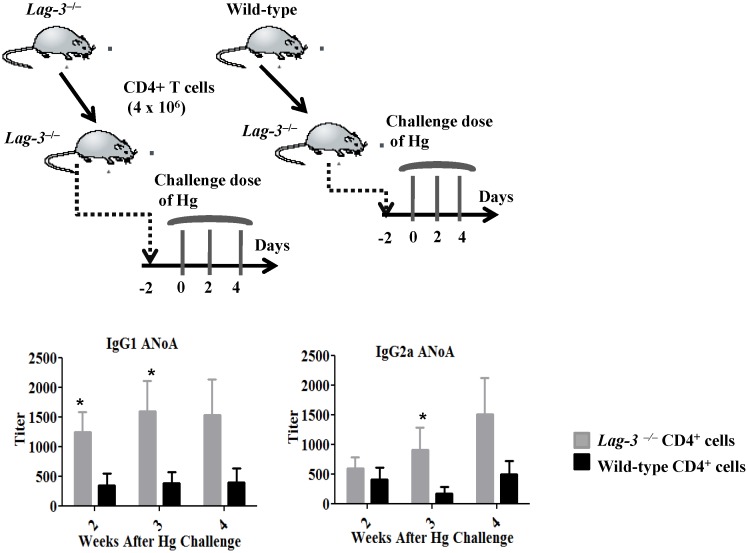
Transfer of wild-type CD4^+^ T cells decreases autoimmunity in LAG-3-deficient mice. CD4^+^ T cells were sorted using FACS and then transferred to LAG-3-deficient mice as depicted in the Figure. Autoantibody titers were measured by IF assay (n = 6). To determine statistical significance between the groups two way ANOVA was used where *indicates *p*<0.05.

Although LAG-3 is expressed on various cell types, CD4^+^ T cells appear to mediate most of its regulatory effects. We therefore hypothesized that transfer of wild-type CD4^+^ T cells might correct the increased susceptibility to autoimmunity in *Lag-3^−/−^* mice (Fig. 7). In this experiment, LAG-3-deficient mice received either 4×10^6^ wild-type CD4^+^ T cells or 4×10^6^ *Lag-3^−/−^* CD4^+^ T cells and were challenged 2 days later with a standard course of 3 HgCl_2_ injections. The wild-type CD4^+^ T cells were able to prevent the induction of autoimmunity as manifested by a significant reduction in titers of IgG1 and IgG2a ANoA following Hg challenge. In addition, serum IgG1 and IgE levels were lower in mice that received wild-type CD4^+^ T cells as compared to the mice that received *Lag-3^−/−^* CD4^+^ T cells, although the levels did not reach statistical significance. Therefore, wild-type CD4^+^ T cells can partially correct the increased autoimmune susceptibility of LAG-3-deficient mice.

## Discussion

The studies in this paper indicate that LAG-3 plays a critical role in decreasing the manifestations of Hg-induced autoimmunity. In the absence of LAG-3-mediated functions, B6.SJL mice that are normally moderately susceptible to the disease become more sensitive to Hg. LAG-3 also plays an essential role in both the induction and maintenance of tolerance to Hg in susceptible animals that can be established via administration of a single low dose of Hg. Hg elicits higher cytokine production in LAG-3-deficient animals and adoptive transfer of wild-type CD4+ T cells can partially rescue LAG-3-deficient B6.SJL mice from Hg-induced autoimmunity.

Our observations support previous studies illustrating that LAG-3 exert a regulatory role upon the immune system. T cell exhaustion occurs during chronic viral infections and results in lack of viral control due to ineffective effector functions of the exhausted antigen-specific CD8+ T cells [36]. Expression of LAG-3 along with other inhibitory receptors such as PD-1 and CTLA-4 is upregulated on exhausted CD8+ T cells during chronic LCMV infection in mice [37], [38]. Dual treatment of mice with blocking antibodies specific for LAG-3 and PD-L1 during chronic LCMV infection resulted in an improved CD8+ T cell response combined with a greater decrease in viral load [38]. In C3HA mice which express hemagglutinin (HA) as self antigen, adoptively transferred HA-specific transgenic CD8+ T (clone 4) cells [39] or HA-specific CD4+ T cells (6.5 T cells) [40] are anergic and show increased expression of LAG-3. Blocking of LAG-3-MHC-II interactions with anti-LAG-3 antibody result in cellular expansion and increased IFN-γ production by clone 4 T cells [39]. Likewise, transfer of Lag-3−/−6.5 T cells causes loss of tolerance and death of recipient C3HA mice [40]. In addition, anergic 6.5 T cells have a higher expression of Foxp3 along with LAG-3 suggesting that tolerized LAG-3+ CD4+ T cells acquire a regulatory T cell phenotype [24], [40]. Taken together, these studies indicate that LAG-3 plays an inhibitory role on immune functions that facilitate persistent viral infections, but allow for the maintenance of self-tolerance. Our current studies demonstrate that mercury-induced autoimmune disease in LAG-3-deficient mice can be partially prevented by adoptive transfer of WT CD4+ T cells, thereby suggesting that LAG-3+ T cells are essential for maintenance of tolerance toward mercury.

Our present study shows that LAG-3 exerts a critical regulatory role in a model of induced experimental autoimmunity. Our results indicate some possible mechanisms by which LAG-3 may exert its effects. Hg-treated LAG-3-deficient B6.SJL mice produced significantly higher amounts of IFN-γ, IL-4 and IL-6 as compared to naïve LAG-3-deficient mice or wild-type mice exposed to HgCl_2_. This is significant since all three cytokines are known to play a critical role in the pathogenesis of Hg-induced autoimmune disease. IFN-γ is responsible for the loss of self-tolerance in this model as IFN-γ-deficient mice fail to produce antinucleolar antibodies in response to Hg [41]. Abrogation of IL-4 functions either by genetic deletion of IL-4 [41], [42] or by *in vivo* administration of a blocking anti-IL-4 monoclonal antibody [43], [44] results in a significant decrease in Hg-induced IgG1 and IgE polyclonal activation [40, 41 and 42]. Likewise, IL-6-deficient mice show a decrease in polyclonal activation following mercury treatment [45], [46]. In addition, our results indicate that LAG-3 expression on CD4^+^ T cells is critical for its regulatory role in Hg-induced autoimmunity since transfer of normal CD4^+^ T cells to LAG-3-deficient mice partially corrects their phenotype.

CD4^+^ T cells that possess a regulatory phenotype display a higher expression of LAG-3 [24], [40], [47]. Tregs deficient in LAG-3 molecule are unable to optimally suppress the autoreactive T cells, whereas ectopic expression of LAG-3 imparts regulatory phenotype to CD4^+^CD25^−^ T cells [17]. Several studies report that Tregs modulate dendritic cell (DC) function and maturation [48]–[50] and Liang et al. have suggested that LAG-3 mediates its effects via DCs [25]. This study showed that LAG-3 expressed on Tregs interacts with MHC-II on DCs and this interaction causes suppression of DC maturation and DC immunostimulatory capacity [25]. The down-regulation of DC function and maturation would then interfere with its ability to stimulate autoreactive effector T cells. Absence of LAG-3 would thus abrogate this Treg-mediated inhibition of DC maturation, eventually culminating in loss of tolerance toward self-antigens. Our preliminary observations suggest that Tregs play an important role in the regulation of Hg-induced autoimmunity [12]. Thus, LAG-3 may mediate its effects via Tregs in preventing autoimmune reactions triggered by Hg.

Although LAG-3 is predominantly expressed on activated T cells and NK cells, a single study reported that B cells can express LAG-3 in a T cell dependent manner [51]. The expression of LAG-3 is limited on activated B cells and it is induced by T cells, thus B cells activated via various other stimuli (bacterial LPS, CD40+ IL-4) do not express LAG-3 [49]. Although both the expression and putative function of LAG-3 on B cells have to be confirmed, its role could be inhibitory as well and could represent an additional regulatory element in Hg-induced autoimmunity, a model which manifests itself essentially via humoral manifestations.

It was recently reported that LAG-3 is constitutively expressed in large amounts on plasmacytoid DCs (pDCs) and that it negatively regulates their activation [52]. CpG-activated *Lag-3^−/−^* pDCs proliferate faster than their wild-type counterparts, although they do not differ in terms of surface molecule expression and cytokine production. Functional studies indicate that LAG-3 on pDCs not only controls the proliferation of pDCs themselves but also regulates the T cell homeostasis [50]. This study suggests that LAG-3 expression on both pDCs and T cells plays a reciprocal regulatory role on the opposite cell type. Although the role of pDCs in Hg-induced autoimmunity remains to be identified, several studies have shown that pDCs in their immature state or in certain anatomical sites have tolerogenic properties [53]–[55]. It is therefore possible that inhibitory effects of LAG-3 on pDCs might have contributed to the observations in the present study.

Taken together, our observations indicate that LAG-3 has an important inhibitory role in the induction and maintenance of tolerance during the course of an induced experimental autoimmune disease. Our study adds autoimmunity to the list of pathological situations such as infections, cancer, and transplantation where LAG-3 can inhibit immune responses. It also suggests that the LAG-3 pathway may represent one of the mechanisms by which tolerance is maintained during autoimmune disease and that restoring or strengthening this pathway could represent a novel therapeutic avenue.
